# An NIR-triggered drug release and highly efficient photodynamic therapy from PCL/PNIPAm/porphyrin modified graphene oxide nanoparticles with the Janus morphology†

**DOI:** 10.1039/c9ra06058h

**Published:** 2019-12-02

**Authors:** Sepideh Khoee, Amirhossein Sadeghi

**Affiliations:** Polymer Laboratory, School of Chemistry, College of Science, University of Tehran PO Box 14155-6455 Tehran Iran khoee@khayam.ut.ac.ir +98 21 66495291 +98 21 61113301

## Abstract

This project aimed to investigate the synthesis and characteristics of stimuli-responsive nanoparticles with different morphologies. In the first step, graphene oxide was synthesized based on the improved Hummers' method. Then, thermo-responsive poly(*N*-isopropylacrylamide-*co-N*-(hydroxymethyl)acrylamide), an amphiphilic copolymer, and poly(caprolactone) (PCL), a hydrophobic polymer, were used to prepare Janus and mixed graphene oxide-based nanoparticles. Fluorescence microscopy was utilized to confirm the Janus structure by labeling the mixed and Janus NPs with fluorescent hydrophobic and hydrophilic dyes *via* a solvent-evaporation method. Then, terminally modified carboxyl porphyrin (TPPC3-COOH), used as the second generation photosensitizer, was grafted to the copolymer surrounding the mixed and Janus NPs. Next, quercetin, a hydrophobic anti-cancer drug, was loaded onto both NPs to accomplish NIR-triggered photodynamic- and chemo-therapy. Finally, the drug loading, encapsulation efficiency, and *in vitro* release of thermo-responsive NPs were investigated at temperatures of 37 °C and 40 °C as well as under laser irradiation (808 nm).

## Abstract 2

This project aimed to investigate the synthesis and characteristics of stimuli-responsive nanoparticles with different morphologies.

## Graphical abstract

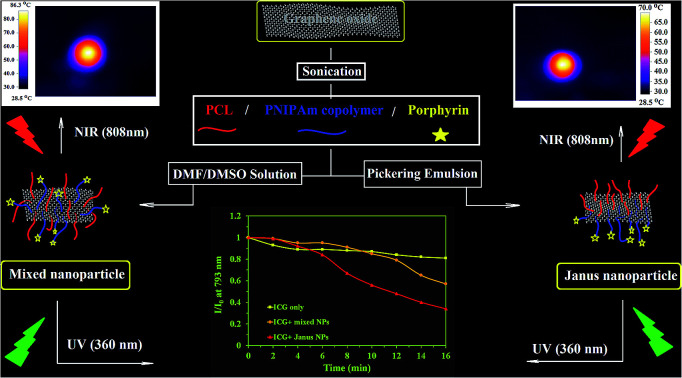

## Introduction

Nanomaterials in different sizes, shapes, and chemical compositions, including metal and metal oxide nanoparticles (NPs), polymeric micelles, liposomes, carbon nanotubes, and carbon nanohorns, have emerged as nanocarriers for the delivery of therapeutic agents.^1–5^ During the past decades, the discovery of graphene has attracted considerable attention and growing interest for use in drug delivery applications. Ideal graphene does not exist naturally, but bulk and solution-processable functionalized graphene materials involving graphene oxide (GO) can now be readily obtained.^6^ Graphene oxide, achieved by the chemical oxidation of graphite, benefits from its tendency to disperse in water and physiological environments owing to the presence of numerous hydrophilic groups, such as hydroxyl, epoxy, and carboxyl groups on its basal surfaces, thus providing options for further functionalization by covalent or non-covalent reactions.^7^ Zhang *et al.* studied the distribution and biocompatibility of GO with an *in vivo* experimental project and reported that GO was rapidly cleared from the bloodstream and delivered to most of the organs over 48 h, but the agglomeration was mainly in the lungs, liver, and spleen, and to a much lesser extent in the brain, heart, and bone. The elimination of GO from the body is based on its size. Accordingly, reducing the size of GO particles addresses the issues with its accumulation in the body.^8^ Without any surface functionalization, GO usually tends to aggregate in water and physiological solutions. Thus, size control and particle separation are required to have adequate interactions with a biological system *in vitro* and *in vivo*.^9–11^ For instance, drug-loaded GO (GO-DTX) was functionalized *via* hydrophobic, π–π, or van der Waals interactions with poly(allylamine hydrochloride) (PAH), which is a cationic, highly water-soluble and biocompatible polymer.^12^ Zhang *et al.* developed a colon-targeted drug delivery system based on alginate–graphene oxide composites. In their studies, GO–ALG/5-FU demonstrated a colon-targeted drug release function, excellent biocompatible properties, and lower drug cytotoxicity.^13^ Recently, dopamine-functionalized graphene oxide was successfully synthesized and loaded with methotrexate (MTX), followed by drug release studies.^14^ An interesting type of polymer for use as the coating is a stimuli-sensitive or smart polymer, also called intelligent polymer, due to its response to external triggers, such as changes in light, temperature, or pH, which has widely been studied in various fields, including tissue culture, enzyme immobilization, and drug delivery systems (DDSs).^15–18^ In the past decades, poly(*N*-isopropylacrylamide) (PNIPAm) has extensively been studied as a thermosensitive polymer in biomedical applications.^19^ The LCST of PNIPAm, which is lower than biological temperatures, can be adjusted to near or above the body temperature by copolymerization with hydrophilic monomers, such as acrylic acid (AA). Therefore, by introducing hydrophilic segments in the polymer backbone, a temperature higher than the LCST is needed for hydrophobic interactions.^20,21^ Recently, amphiphilic Janus NPs with bifacial architectural features have appeared as a new kind of colloidal structure to make drug delivery systems more efficient. Various methodologies have been applied to prepare Janus NPs with different functions, such as Pickering emulsion methods, block copolymer self-assembly, microfluidics, and electro co-jetting.^22–26^

Recently, photodynamic therapy (PDT) has become a noticeable protocol in the treatment of cancer and in the inactivation of microorganisms and viruses.^27,28^ In this method a photosensitizer (PS) is introduced into cancer cells irradiating with a laser or visible light, thus generating molecular oxygen.^29–31^ The reactive oxygen species (ROS) generated by photosensitizers can kill cancer cells by destroying DNA, RNA, lipids, and proteins.^32^ However, PDT still has limitations, such as nonspecific tissue biodistribution, insufficient cancer accumulation, rapid renal clearance, and other adverse effects. Porphyrins, a group of organic compounds with a macrocycle conjugated structure, are used as second generation PSs in PDT. Porphyrins and their derivatives exhibit desirable properties, such as selectivity for particular diseased tissues and comparatively fast elimination from the body. However, most porphyrins and their derivatives have hydrophobic properties, making them undispersed and unstable in an aqueous medium, which is a considerable limitation for therapeutic applications. Also, most PSs tend to aggregate in aqueous solution, in which the π–π stacking greatly decreases the yield of ^1^O_2_ and therefore, the therapeutic efficiency.^33^ To address these issues, much effort has been made to enhance the properties of porphyrin PSs. For example, Zhang *et al.* prepared reduction-sensitive nanomedicines from porphyrin-containing small molecules and improved the photodynamic therapeutic efficacy at the cellular level.^27^ Li *et al.* constructed porphyrin-based carbon dots (TPP CDs) from mono-hydroxylphenyl triphenylporphyrin (TPP) and chitosan, which showed good water solubility and excellent quantum yields of singlet oxygen.^34^

In this work, to control singlet oxygen generation by precise localization and achieve enhanced retention in cancer cells, a system was designed with a more efficient PDT and fewer side effects. To this end, we synthesized mixed and Janus graphene oxide NPs by covalently attaching different types of polymers on their two faces. Poly(*N*-isopropylacrylamide-*co-N*-(hydroxymethyl)acrylamide), synthesized by the ATRP method, and poly(caprolactone) were used to functionalize GO NPs through a Pickering emulsion of oil in water (o/w) and a homogeneous solution. To prepare NPs with the ability to generate singlet oxygen (^1^O_2_), these mixed and Janus NPs were decorated with TPPC3-COOH as the second generation photosensitizers. Finally, quercetin, a hydrophobic anti-cancer drug, was loaded in these NPs, and the *in vitro* release profile of quercetin was studied in a phosphate buffer solution at two temperatures of 37 and 40 °C (pH = 7.4), as well as under laser irradiation.

## Experimental

### Materials

Graphite flakes, phosphoric acid (H_3_PO_4_), potassium permanganate (KMnO_4_), *N*,*N*-dicyclohexylcarbodiimide (DCC), 4-dimethylaminopyridine (DMAP), copper bromide (CuBr), ε-caprolactone, *N*-isopropylacrylamide (NIPAm), and *N*-methylolacrylamide (NMA) were all purchased from Merck Chemical Co. Moreover, *N*-hydroxysuccinimide (NHS), *N*-ethylcarbodiimide hydrochloride (EDC), pyrrole, succinic anhydride, 3-bromo-1-propanol, stannous octoate (Sn(Oct)_2_), and *N*,*N*-dimethylformamide (DMF) were all purchased from Aldrich. All the chemicals were of analytical grade and used without any purification.

### Characterization and equipment

Characterization methods and equipment are reported in the ESI.†

### Synthesis of graphene oxide

Graphite flakes were oxidized based on an improved Hummers' method.^35^ Briefly, a mixture of graphite powder (1 g) in 200 mL of an acid mixture of H_2_SO_4_ and H_3_PO_4_ (9 : 1, v/v) was prepared under non-stop stirring conditions. Then, KMnO_4_ (6 g) was gradually added to the mixture and the reaction was continued for three days at 50 °C. After the completion of the reaction, the mixture was allowed to become cold and then poured into ice water. H_2_O_2_ (4 mL) was added dropwise to the cold mixture under constant stirring until the color of the solution changed to yellow. Afterward, the mixture was centrifuged and the precipitate was washed twice with an HCl aqueous solution (v/v, 10%) and three times with distilled water. Dried graphene oxide was obtained after lyophilization.

### Synthesis of *tert*-butyl-*N*-(2-aminoethyl)carbonate (1)

According to ref. 36, a solution of 13.1 g (0.06 mol) di-*tert*-butyldicarbonate in 100 mL of 1,4-dioxane was gradually poured into a solution of 27.8 g (0.46 mol) of diaminoethane in 150 mL of 1,4-dioxane over 3 h at room temperature. After 48 h, the precipitate was filtered off and 1,4-dioxane and extra diaminoethane were removed under vacuum. Distilled water (250 mL) was poured into the residue solution and bis(*N*,*N*′-*tert*-butyloxycarbonyl)-1,2-diaminoethane was removed by filtration. Saturated sodium chloride was added to the solution and extracted with dichloromethane (4 × 100 mL). The organic phase was dried with anhydrous magnesium sulfate, and dichloromethane was evaporated under reduced pressure. Finally, *tert*-butyl-*N*-(2-aminoethyl)carbonate was obtained as a colorless oil (90% yield).

### Synthesis of *tert*-butyl-2-(2-bromo-2-methylpropanamido)ethyl carbamate (2)

*tert*-Butyl-*N*-(2-aminoethyl)carbonate 1 (3.6 g, 22.50 mmol) was dissolved in 40 mL of THF in the presence of trimethylamine (3.5 g, 33 mmol), and then 2-bromoisobutyryl bromide (7.77 g, 33 mmol) was slowly added to the solution in an ice bath. The reaction solution underwent non-stop stirring at room temperature for 48 h. Triethylammonium bromide as a white precipitate was filtered off and, after the removal of the solvent under vacuum, a yellow solid was obtained which was then dissolved in methanol and precipitated into water saturated with Na_2_CO_3_. Compound 2 was obtained with a 95% yield.^36^

### Synthesis of P(NIPAm-*co*-NMA)

The P(NIPAm-*co*-NMA) was prepared *via* atom transfer radical polymerization (ATRP), and 2 was used as an ATRP initiator. First, NIPAm (538.65 mg, 4.76 mmol), NMA (35.34 mg, 0.35 mmol), and CuBr (6.88 mg, 0.048 mmol) were dissolved in methanol/water (3 : 2, the total volume of solution is 5 mL) at room temperature. The initiator (15.25 mg, 0.049 mmol) and PMDETA (20.7 μL) were separately dissolved in 2 mL of methanol/water (3 : 2) and poured into the solution. The solution was purged with nitrogen and heated at 60 °C for 8 h. To quench the polymerization, the reaction mixture was exposed to air for 2 h. Then, 4 mL of tetrahydrofuran (THF) was added to the polymerization solution and the final solution was filtered over alumina to remove the catalyst. Next, the solvent was removed by vacuum evaporation and freeze-dried, respectively. P(NIPAm-*co*-NMA) was obtained as a white precipitate and then dissolved in THF and precipitated in hexane. This cycle was repeated in triplicate to achieve a pure product.

### Synthesis of poly(ε-caprolactone) (PCL)

According to a previously described method,^37^ poly(ε-caprolactone) was synthesized using ethanol as an initiator, with slight modification. Briefly, a solution of ε-caprolactone (2 mL, 18.75 mmol) in DMF (5 mL) was placed in a three-necked round-bottom flask under nitrogen atmosphere. Then, ethanol (0.15 mL, 2.50 mmol) was poured into the reaction mixture and the temperature was gradually raised to 80 °C. Afterward, a catalytic amount of Sn(Oct)_2_ was added to the mixture and the solution temperature was adjusted at 120 °C. The reaction medium was allowed to continue at this temperature for 24 h. The raw product was purified by dissolving in DMF and then precipitating in cold water. This cycle was repeated twice to obtain a pure product.

### Synthesis of 5-(4-hydroxylphenyl)-10,15,20 triphenylporphyrin (TPP-OH)

A solution of 4-hydroxybenzaldehyde (780 mg, 6.39 mmol) in 50 mL of propionic acid was heated to reflux at 140 °C. Benzaldehyde (2 mL, 19.74 mmol) was then added to the reaction mixture. After 15 min, a solution of pyrrole (1.74 mL, 25.05 mmol) in 5 mL of propionic acid was added dropwise, and the mixture was refluxed for another 45 min. After cooling to room temperature, 10 mL of methanol was poured into the mixture and stored in the refrigerator overnight. A purple solid was obtained by washing with cool methanol. The raw product was purified by column chromatography (silica gel) using chloroform as the eluent (yield = 30%).^38^

### Synthesis of 3-(5′-(4′-phenoxyl)-10′,15′,20′-triphenylporphyrin)-1-propanol (TPPC3-OH)

According to ref. 38, with a few changes, TPPC3-OH was synthesized from TPP-OH and 3-bromo-1-propanol. Potassium carbonate (40 mg, 0.29 mmol) was added to a solution of 3-bromo-1-propanol in 5 mL of DMF. The reaction mixture was refluxed for 16 h and washed with distilled water three times. The residue was dissolved in CH_2_Cl_2_ and dried with anhydrous MgSO_4_. Dichloromethane was then removed under vacuum, and the crude product purified with column chromatography (silica gel) using a mixture of petroleum ether–ethyl acetate (4 : 1) as the eluent (yield = 90%).

### Synthesis of terminal modified carboxyl porphyrin (TPPC3-COOH)

TPPC3-COOH was synthesized by an esterification reaction between TPPC3-OH and succinic anhydride. Succinic anhydride (520 mg, 5.2 mmol) and pyridine (catalytic amount) were dissolved in 25 mL of anhydrous methylene chloride at room temperature. Then, DMAP (60 mg, 0.49 mmol) was added to the solution in an ice bath. TPPC3-OH (300 mg, 0.43 mmol) was mixed in 15 mL of methylene chloride and added dropwise to the solution. The reaction was performed at 25 °C for 48 h, and then the solution was washed by water saturated with NaCl and extracted with dichloromethane. The organic phase was dried with anhydrous MgSO_4_ and the solvent was removed under vacuum (yield = 90%).

### Preparation of NPs

#### Synthesis of Janus poly(ε-caprolactone)-graphene oxide-poly(NIPAm-*co*-NMA) (J-(PCL-GO-copolymer))

The Pickering emulsion of the oil-in-water (o/w) method was used to prepare J-(PCL-GO-copolymer). To this end, a solution of PCL (20 mg), DCC (8.5 mg), and DMAP (2.5 mg) in 2 mL DCM as an oil phase was poured dropwise into the homogeneous colloidal suspension prepared beforehand through the dispersion of 10 mg of GO in 10 mL of phosphate buffer saline (pH adjusted to 7.4). The micro-emulsion of o/w appeared under a high-rate and non-stop stirring. The first reaction was stopped after 4 h to confirm the formation of J-(PCL-GO) through several analyses. After that, solid J-(PCL-GO) was dispersed once more in PBS (10 mL) and sonicated for 30 min (2 s on/2 s off) to obtain a homogeneous colloidal suspension of J-(PCL-GO). NHS (20 mg) and EDC (30 mg) were added gradually to the reaction solution and stirred for 2 h at room temperature. Then, poly(NIPAm-*co*-NMA) (50 mg) was added to the reaction, and the mixture was kept at room temperature for 16 h. The aqueous phase was evaporated by the freeze-dryer, and the residue was washed with DCM and then with water to remove unnecessary substances.

#### Synthesis of mixed poly(ε-caprolactone)-graphene oxide-poly(NIPAm-*co*-NMA) (m-(PCL-GO-copolymer))

To prepare m-(PCL-GO-copolymer), GO (10 mg) was dispersed in DMSO/DMF (1 : 1, the total volume is 10 mL) through an ultrasound probe (15 min, 45%). Then, PCL, copolymer, DCC, and DMAP were simultaneously added to the GO suspension. The reaction was continued for 24 h at room temperature. Afterward, the solvent was evaporated through the freeze-dryer, and the product was washed with water and DMF and dried overnight under vacuum.

#### Functionalization of J-(PCL-GO-copolymer) and m-(PCL-GO-copolymer) by TPPC3-COOH

TPPC3-COOH grafted to Janus and mixed NPs was obtained through the following steps: briefly, J-(PCL-GO-copolymer) (10 mg) was dispersed in 10 mL of DMSO/DMF (4 : 1) with the ultrasonic probe (20 min, 45%). Then, TPPC3-COOH (4.5 mg), the photosensitizer, DMAP (2.5 mg), and DCC (8.5 mg) were added to the colloidal suspension, and the mixture was left to continue for 72 h under non-stop stirring. Next, the solvent was evaporated by the freeze-dryer. In the final step, the Soxhlet extractor was used for 48 h to remove the redundant porphyrins, DCC, and DMAP. Mixed-(PCL-GO-copolymer) containing porphyrin was also prepared with the same procedure.

### Preparation of drug-loaded NPs

First, 10 mg of the mixed and Janus NPs were separately sonicated in 10 mL of DMSO/DMF (4 : 1). Then, quercetin (1 mg), a hydrophobic anti-cancer drug, was added to each colloidal suspension. The mixture was left to continue for 48 h at room temperature. Drug-loaded NPs were obtained after lyophilization and washed once with the solvent to remove the unloaded drug. In the final step, dried NPs were achieved after freeze-drying.

UV-Vis spectroscopy was used to calculate the drug loading and encapsulation efficiency of both NPs. A distinct amount of drug-loaded NP was dispersed in DMSO/DMF (4 : 1) to measure the trapped drug. The solvent mixture of DMSO and DMF easily solvated both PCL and the copolymer and, as a result, the encapsulated quercetin abruptly diffused into the solvent medium. Then, a high-speed centrifuge was employed to separate the insoluble NPs, and the absorbance of quercetin was determined by a UV-Vis spectrophotometer at its maximum absorption wavelength (370 nm), which was assessed with the help of a pre-determined standard calibration curve of the drug in DMSO/DMF (4 : 1). The following equations (eqn (1) and (2)) were utilized to measure the drug loading (DL) and encapsulation efficiency (EE):1
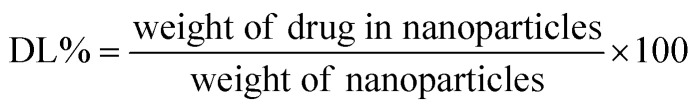
2
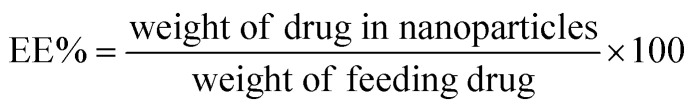


### *In vitro* release of quercetin from NPs

An equal amount of the quercetin-loaded mixed and Janus NPs (1 mg) was separately dispersed in a phosphate buffer saline (pH = 7.4) and transferred to a dialysis bag (molecular weight cut-off of 12 kD). The bag was placed into a flask containing 50 mL of fresh PBS (pH = 7.4) and stirred at the rotating speed of 150 rpm. The experiment was conducted at both temperatures of 37 and 40 °C. Next, 2 mL of the release medium was taken at distinct time intervals and replaced with fresh PBS. The amount of released quercetin was calculated through UV-Vis spectroscopy at the wavelength of 203 nm. The drug release percentage was determined according to eqn (3).3
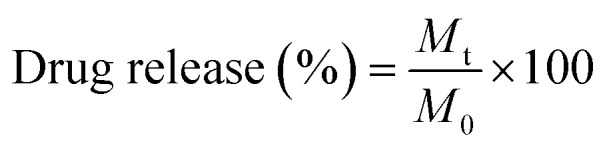
*M*_0_ and *M*_t_ are related to the amount of loaded and released drug, respectively.

### Laser irradiation of drug-loaded mixed and Janus NPs

To evaluate the photothermal effect of NPs, an equal amount of the quercetin-loaded mixed and Janus NPs was irradiated for 20 min by an 808 nm continuous-wave NIR laser (power density of 2 W cm^−2^ and spot size of 2 cm^2^). During the NIR exposure, temperature changes were monitored by a K thermocouple (nickel–chromium) linked to a digital thermometer (Lutron Thermometer TM-917, Taiwan) every 2 min for 20 min.

### Singlet oxygen detection

A chemical method using ICG (indocyanine green) was applied to assess the capability of the porphyrin-grafted mixed and Janus NPs in singlet oxygen generation. First, a mixture of 80 μL of a colloidal suspension of porphyrin-grafted Janus NPs in DMF (1 mg mL^−1^) and 10 μL of ICG water solution (1 mg mL^−1^) was added into DMF (10 mL) and irradiated by a UV light source (360 nm, mW cm^−2^). Then, UV-Vis spectroscopy was utilized to detect the absorption intensity of the solution every 2 min. The same procedure was replicated for mixed NPs to assess the ability of singlet oxygen generation under irradiation.

### Cell cytotoxicity assay

The relative cell viability was measured in the presence of quercetin, Janus and mixed NPs, quercetin-loaded Janus and mixed NPs, rat C6 glioblastoma and OLN-93 cells (rat brain non-tumor cell line) *via* conventional 3-(4,5-dimethylthiazol-2-yl)-2,5-diphenyltetrazolium bromide (MTT) assays. The cells with an initial seeding density of 5 × 10^3^ cells per well were seeded in 96-well plates and cultured for 24 h. For the *in vitro* phototoxicity of various samples, a cell culture was removed and cells were treated with 100 μL fresh medium containing either the above-mentioned samples at the different concentrations for another 24 h. After replacing the fresh culture medium, the cells were exposed to irradiation with a halogen lamp (500–800 nm, 70 mW cm^−2^) for 10 min. Then, the plates were incubated for another 24 h. 1 mM freshly-prepared MTT was added to each well and the samples were incubated for 4 h. Then, the culture media were removed and 100 μL DMSO was added to each well. Prepared samples were incubated for 10 min. The absorption of the obtained solution was measured at 540 nm. For the dark cytotoxicity, the same procedure and conditions were used but without illumination.

### Statistical analysis

Statistical analysis of all experiments was done in three independent tests. Results were reported as the mean ± SD. All statistical analyses were carried using the software Graph Pad Prism 6. The data comparing was performed using a one-way ANOVA. *P* < 0.05 shows the statistical significance.

## Results and discussion

### Synthesis and characterization of polymer-grafted GO NPs with two morphologies

**Scheme 1:**
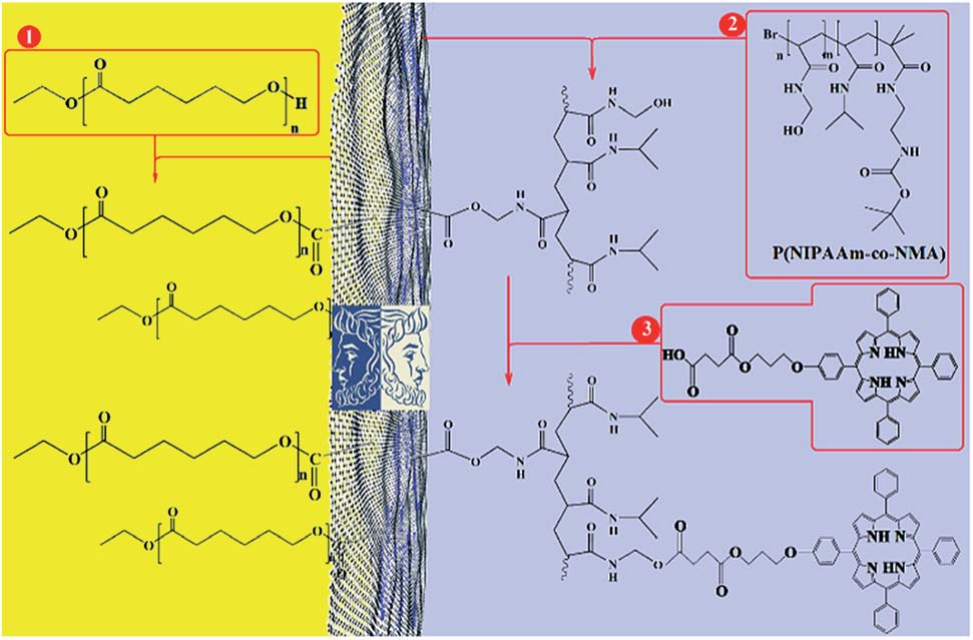
Total synthesis of porphyrin-decorated Janus poly(ε-caprolactone)-graphene oxide-poly(NIPAm-*co*-NMA).

This paper presents two methods for the functionalization of GO NPs with two polymers. First, GO NPs were prepared based on the improved Hummer's method. Then, PCL and P(NIPAm-*co*-NMA) were grafted to both or either side of the GO sheets in the absence of emulsifier with two different methods. Finally, mixed and Janus nanoparticles were functionalized with TPPC3-COOH (Scheme 1).

**Fig. 1:**
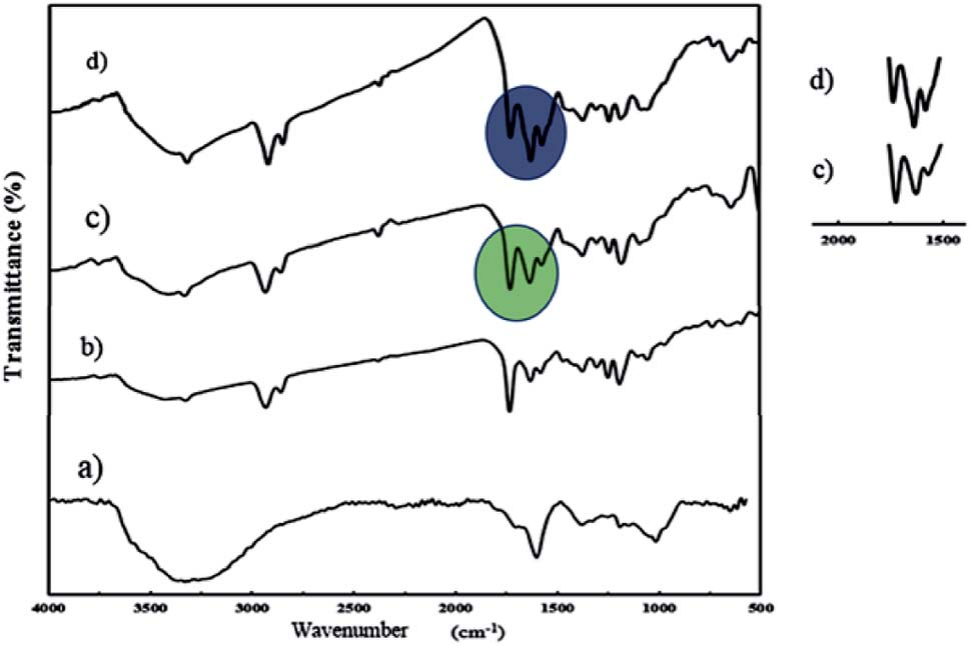
FT-IR spectra of GO (a), J-(PCL-GO) (b), J-(PCL-GO-copolymer) (c), and m-(PCL-GO-copolymer) (d).

An ultrasonic probe was utilized to decrease the size of GO sheets from millimeters to nanometers. Moreover, GO NPs were synthesized by the oxidation of graphite flakes through the improved Hummer's method (Scheme S1†). The FT-IR spectrum of GO NPs is depicted in Fig. 1a, and the broad peak at 3300–3500 cm^−1^ was assigned to the stretching vibration of the OH group. Also, the peaks at 1725, 1626, and 1223 cm^−1^ were attributed to the C=O, C=C, and C–O stretching of the carboxylic, aromatic hydrocarbon, and epoxy groups present on the surface of the GO NPs, respectively.

**Scheme 2:**
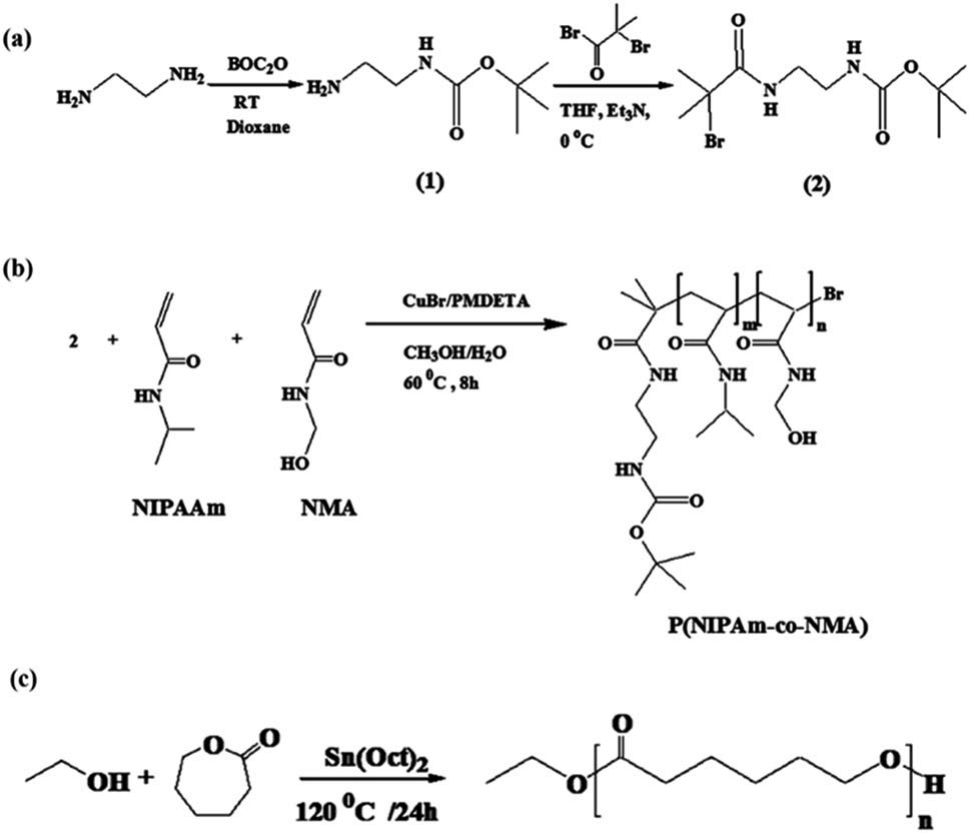
Synthesis of initiator (a), P(NIPAm-*co*-NMA) (b), and PCL.

The synthetic procedures used for the synthesis of P(NIPAm-*co*-NMA) are described in Scheme 2.

**Fig. 2:**
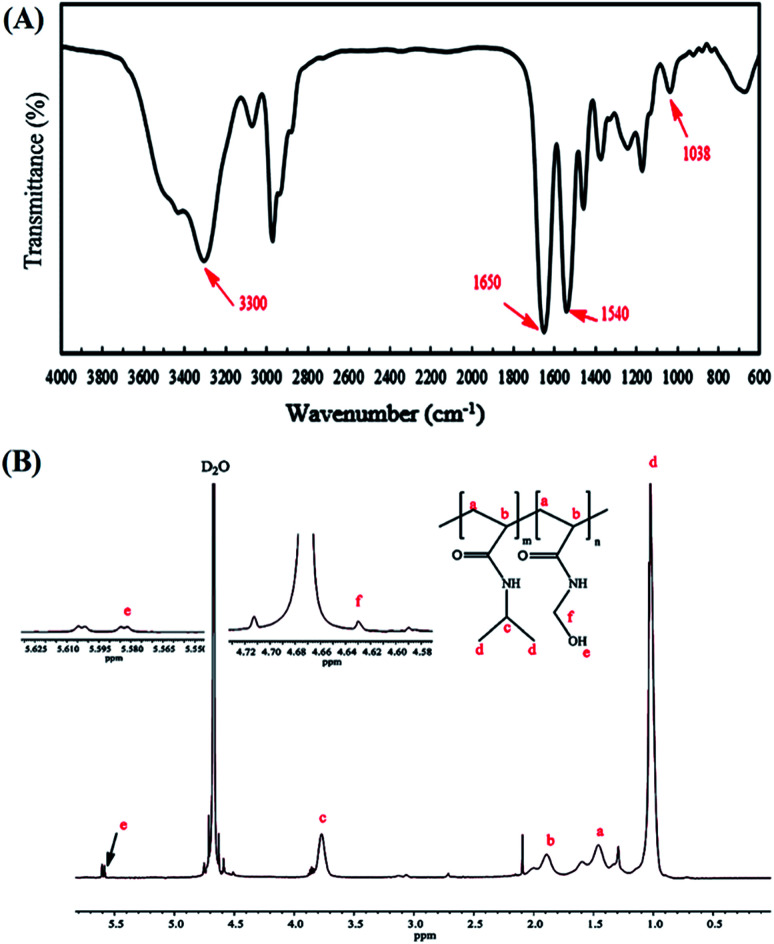
FT-IR (A) and ^1^H NMR (in D_2_O) (B) spectra of poly(NIPAm-*co*-NMA).

The copolymer of NIPAm and NMA was easily prepared by the ATRP method using *tert*-butyl-2-(2-bromo-2-methylpropanamido)ethyl carbamate (2) as the initiator. The ^1^H NMR spectrum of the initiator shows the methyl proton resonance peaks (s, 9H, C(CH_3_)_3_ at *δ* = 1.47 ppm and s, 6H, C(CH_3_)_2_ at *δ* = 1.97 ppm), along with methylene protons (q, 4H, CH_2_CH_2_) at *δ* = 3.37 ppm and amine protons (s, 2H, NH) at *δ* = 4.89 ppm in CDCl_3_ (Fig. S1†). The FT-IR spectra of P(NIPAm-*co*-NMA) are illustrated in Fig. 2A.

The characteristic peaks of the copolymer appearing at 1540 and 1650 cm^−1^ were attributed to the C=O stretching and N–H bending of the copolymer, respectively. Furthermore, the successful polymerization of the monomers was confirmed by the disappearance of the absorption band at 1655 cm^−1^, which are associated with the C=C in the vinyl groups. The peaks at 1038 and 3300–3500 cm^−1^ were assigned to the C–O–H and O–H stretching in NMA, respectively. The FT-IR spectrum also shows the main chain absorption band at 2971 cm^−1^ assigned to the C–H stretching bond in the copolymer. The chemical structure of p(NIPAm-*co*-NMA) was also investigated by ^1^H NMR spectroscopy in deuterated water (D_2_O) (Fig. 2B). The signals at *δ* = 1.35–2.5 ppm (a and b) were assigned to the methylene protons (–CH_2_CH–) of NMA and NIPAm. Furthermore, the peaks at 1.02 (d) and 3.77 ppm (c) were related to NIPAm (–CH(CH_3_)_2_) and NIPAm (–CH(CH_3_)_2_), respectively. The signals at 4.63 (f) and 5.58 ppm (e) were associated with (–NHCH_2_–OH) and (–CH_2_OH) for NMA, respectively.^39^ Poly(*N*-isopropylacrylamide) and its copolymers act as thermo-responsive polymers. The LCST of PNIPAm is around 32 °C. It is argued that the LCST of PNIPAm can be adjusted to the body temperature (37 °C) *via* copolymerization with hydrophilic monomers.^40^ Therefore, poly(NIPAm-*co*-NMA), with the molar ratio of (13.5 : 1), was synthesized to increase the LCST of pure PNIPAm. A UV-Vis spectrophotometer equipped with a temperature controller was utilized to measure the cloud point value of the copolymer solution in water (Table 1). Based on Fig. S2†, the cloud point of poly(NIPAm-*co*-NMA) shifted to 38.5 °C, indicating that NMA had a significant effect in the copolymer. The molecular weight and the actual NMA content (eqn (4)) in the copolymer were calculated by ^1^H NMR spectroscopy.4
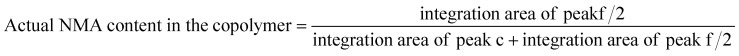


**Table 1:** Information on the NIPAm-NMA random copolymer

Sample	Initial feed molar ratio (NIPAm : NMA)	Actual NMA content^a^ (%)	Mn^b^	CP^c^ (°C)
P(NIPAm-*co*-NMA)	13.5 : 1	4.76	11 500	38.5

a The molar content of NMA in poly(NIPAm-*co*-NMA) determined by ^1^H NMR.

b The molecular weight of the polymer calculated by ^1^H NMR.

c The cloud point measured by UV-Vis spectroscopy.

The NMA content was estimated by the integration of CH(CH_3_)_2_ (3.77 ppm) for NIPAm and NH–CH_2_–OH (4.63 ppm) for NMA. Table 1 presents the detailed information of p(NIPAm-*co*-NMA).

Poly(ε-caprolactone) was prepared by the ring-opening polymerization of ε-caprolactone in the presence of ethanol and Sn(Oct)_2_ acting as the initiator and catalyst, respectively, at 120 °C (Scheme 2C). Fig. S3† depicts the FT-IR spectrum of PCL. The characteristic peaks of PCL appeared at 1725 and 1189 cm^−1^, each associated with the C=O and C–O stretching bonds of the ester group. Furthermore, the peak at the 2942 cm^−1^ region was assigned to the C–H stretching in the PCL chain. The ^1^H NMR spectrum of PCL shows six major resonance peaks (Fig. S4†). The triplet and multiplet peaks at 0.9 ppm (a) and 4.23 ppm (b) were attributed to the methyl and methylene protons of ethanol, respectively. The methylene protons of the PCL chain appeared at 1.25 (e), 1.7 (d), 2.2 (c), and 4.15 (f) ppm. Moreover, the average molecular weight of PCL is 2000 g mol^−1^, obtained by ^1^H NMR spectroscopy.

**Scheme 3:**
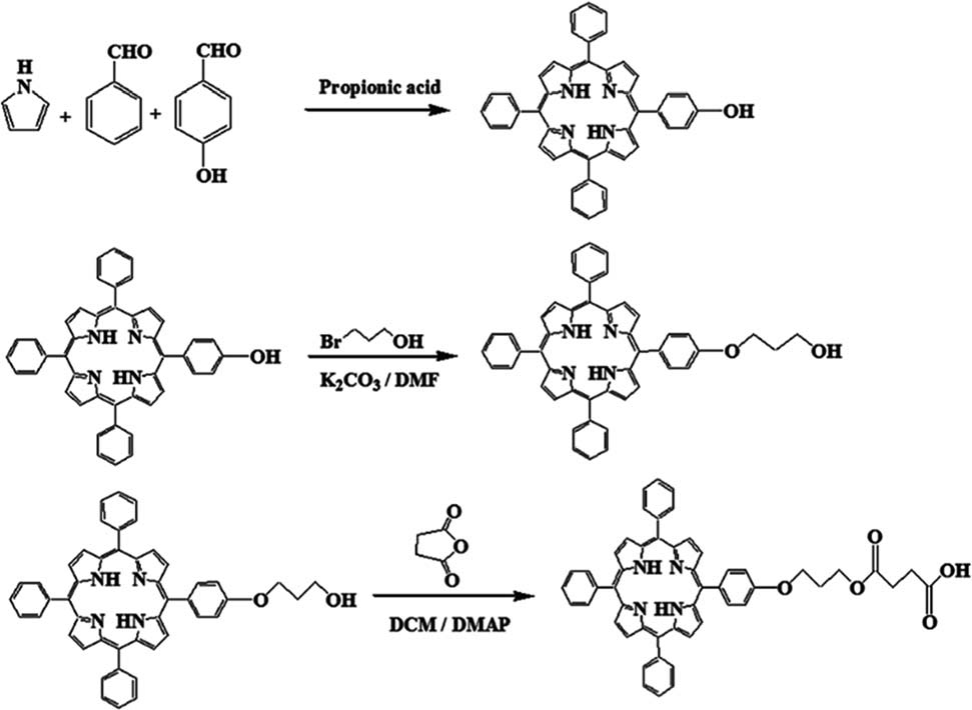
Synthesis of TPPC3-COOH.

**Fig. 3:**
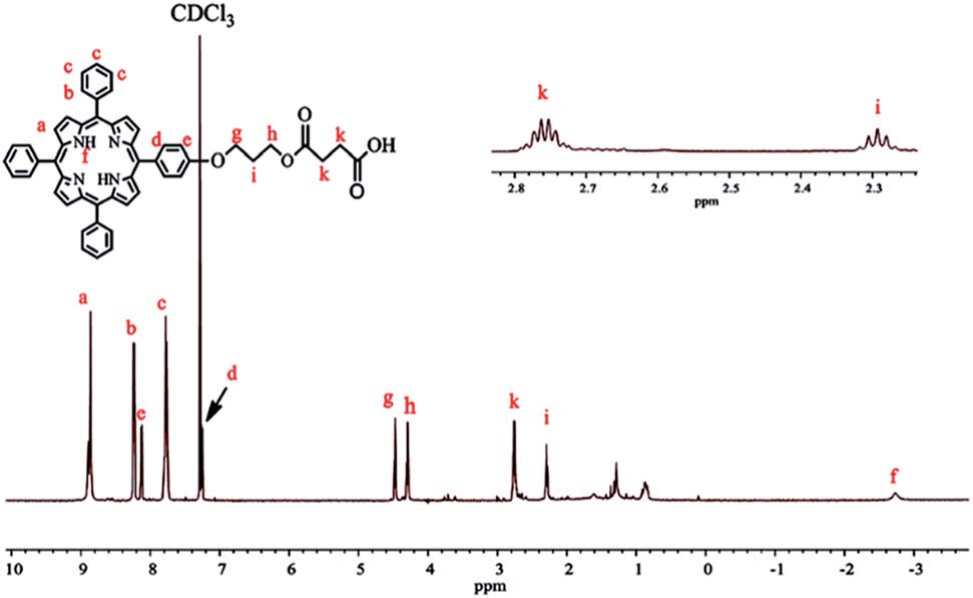
^1^H NMR spectrum of TPPC3-COOH in CDCl_3_.

To prepare TPPC3-COOH (Scheme 3), TPP-OH was first synthesized and characterized by ^1^H NMR (Fig. S5†). 6-Chloro-1-hexanol is the most common spacer for the functionalization of TPP-OH.^27,38,41^ However, in the present work, the hydroxyl group of TPP-OH was expanded to produce TPPC3-OH using 3-bromo-1-propanol for the first time because a much shorter spacer affects the solubility of the porphyrin derivatives to a lesser extent. The ^1^H NMR spectrum of TPPC3-OH is shown in Fig. S6†. The signals at *δ* = 8.86–7.25 ppm and −2.73 ppm were ascribed to the aromatic and pyrrole ring protons separately, and the signals at 4.43, 4.05 and 2.25 ppm were assigned to the methylene protons. Finally, TPPC3-COOH was synthesized for the first time *via* an esterification between TPPC3-OH and succinic anhydride. The structure of TPPC3-COOH was determined by ^1^H NMR spectroscopy in CDCl_3_ (Fig. 3) and a new peak appeared at *δ* = 2.75 ppm, which belonged to the methylene protons (–CO–CH_2_–CH_2_–CO–), indicating that TPPC3-COOH was successfully synthesized.

Moreover, the UV-Vis absorption of TPPC3-COOH (Fig. S7†) exhibits an intense peak at 420 nm (Soret band, a characteristic absorption of the electronic spectrum of porphyrin) and a less intense peak at 548 nm known as the Q band.

J-(PCL-GO-copolymer) NPs were synthesized with the Pickering emulsion of o/w method. P(NIPAm-*co*-NMA) possessed amphiphilic behavior and could diffuse into the organic phase. To prevent this problem, J-(PCL-GO-copolymer) NPs were prepared in two steps. Briefly, the oil phase containing the solution of PCL in DCM was added dropwise into the colloidal suspension of GO in phosphate buffer saline (PBS) to obtain J-(PCL-GO). The FT-IR spectrum of J-(PCL-GO) NPs (Fig. 1b) has a strong peak at 1728 cm^−1^, related to the C=O stretching bond of the ester group in PCL. In comparison with the FT-IR spectrum of GO NPs (Fig. 1a), the appearance of PCL patterns in the FT-IR spectrum of J-(PCL-GO) NPs confirmed the successful graft of PCL on the surface of the graphene oxide. In the second step, p(NIPAm-*co*-NMA) was added to the colloidal suspension of J-(PCL-GO) in the PBS medium to prepare J-(PCL-GO-copolymer) NPs. Thus, there would be a clear boundary between the two polymers grafted on the surface of graphene oxide NPs. The FT-IR spectrum of J-(PCL-GO-copolymer) NPs is shown in Fig. 1c. Compared to J-(PCL-GO), the peaks at 1630 and 1570 cm^−1^ became stronger, related to the C=O stretching and N–H bending vibration of the copolymer, respectively.

A one-step reaction was designed to synthesize mixed (PCL-GO-copolymer) NPs. Graphene oxide was sonicated in a mixture solvent of DMSO/DMF (1 : 1) and then both polymers were simultaneously added to the suspension. In this way, PCL and p(NIPAm-*co*-NMA) were haphazardly grafted on both sides of the graphene oxide NPs. The FT-IR spectrum of mixed NPs (Fig. 1d) illustrates that all the characteristic peaks belonging to the functional groups grafted on the surface of graphene oxide NPs are present. Compared to the Janus NPs, the weak peak at 1728 cm^−1^ (related to the C=O stretching vibration in PCL) appeared in the spectrum of mixed NPs, attributed to the interactions between PCL and copolymer. However, the peaks at 1728 and 1631 cm^−1^ in the Janus spectrum become more distinct and stronger than the identical peaks in the spectrum of mixed NPs, demonstrating that the PCL-copolymer interactions are not significant in the Janus NPs.

**Fig. 4:**
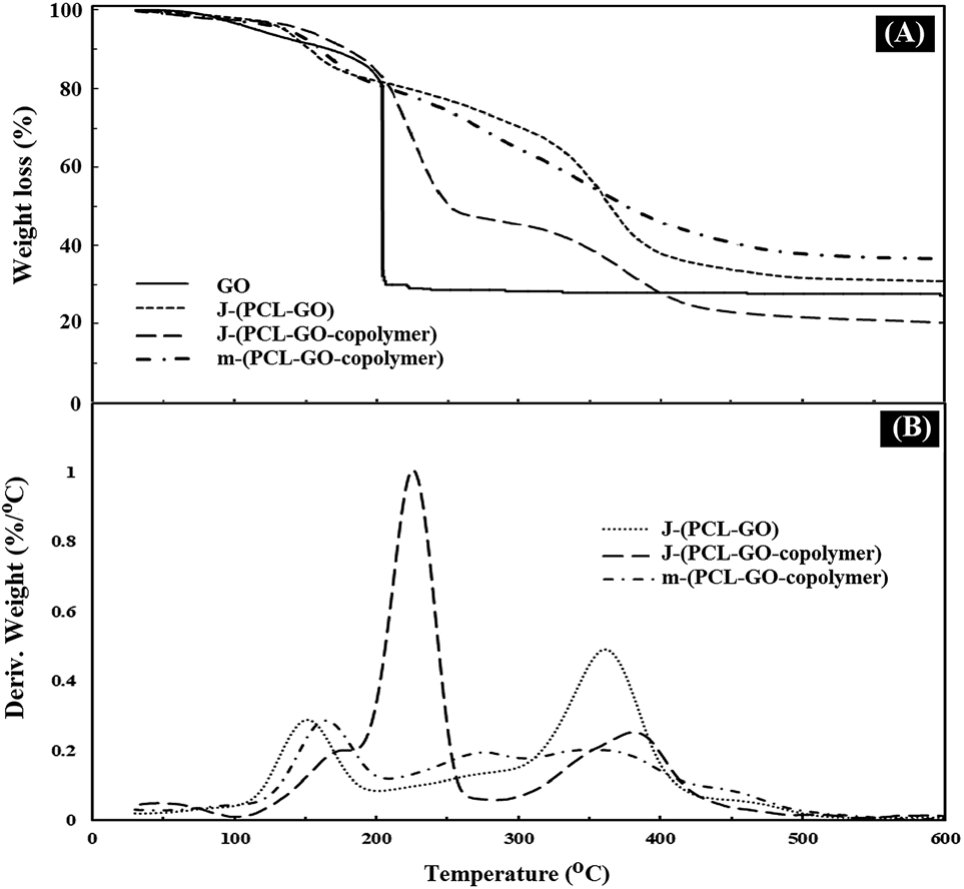
TGA (A) and DTG (B) thermograms of GO, J-(PCL-GO), J-(PCL-GO-copolymer), and m-(PCL-GO-copolymer).

TGA analysis was used to measure the percentage of polymers grafted on the surface of the mixed and Janus NPs. The weight loss curves of GO (a), J-(PCL-GO) (b), J-(PCL-GO-copolymer) (c), and m-(PCL-GO-copolymer) (d) are presented in Fig. 4. Pure graphene oxide showed a weight loss of about 67%, related to the degradation of unstable oxygen-containing functional groups as well as water evaporation. The weight loss curve of J-(PCL-GO) (Fig. 4A and B) depicted two distinct regions. The first mass loss (18%) was observed before 200 °C, which is related to the degradation of GO, and the second one (50%) appeared after 220 °C, which is related to the decomposition of PCL grafted onto the surface of J-(PCL-GO) NPs. According to the TGA curves, J-(PCL-GO-copolymer) NPs gradually lost 15% of their total weight before 200 °C, which is attributed to the destruction of GO. Then, a weight loss of about 40% was observed from 200 to 290 °C, belonging to the decomposition of p(NIPAm-*co*-NMA). Finally, the third weight loss (25%) from 310 to 600 °C was attributed to the degradation of PCL, verifying the composition of the J-(PCL-GO-copolymer) NPs. m-(PCL-GO-copolymer) NPs lost 20% of their total weight before 200 °C, which is attributed to the destruction of unstable functional groups on the surface of the graphene oxide. Then, a mass loss of 15% appeared from 210 to 300 °C, related to the degradation of the copolymer on the mixed NPs. Finally, a larger weight loss of about 28% was observed from 310 to 600 °C, which is attributed to the decomposition of PCL in the sample. The TGA measurement of J-(PCL-GO-copolymer) NPs in comparison to that of the mixed NPs showed a distinct gap between the decomposition of PCL and copolymer due to the lack of interactions between them in the Janus NPs. This observation is consistent with the findings of previous scientists who demonstrated that this two-stage weight loss is more clearly defined for the block copolymers with micro-phase-separated domains in comparison that of random ones.^42^ Also, the TGA curves demonstrated that the mixed NPs have a higher thermal stability than that of the Janus ones, and additional energy is required for the degradation of further-mixed NPs. The reason for this is that the polymer–polymer interactions in the mixed NPs lead to a lower chain mobility and higher hydrogen bonding. Moreover, no distinct peaks were observed for the two polymers in the mixed sample.

**Fig. 5:**
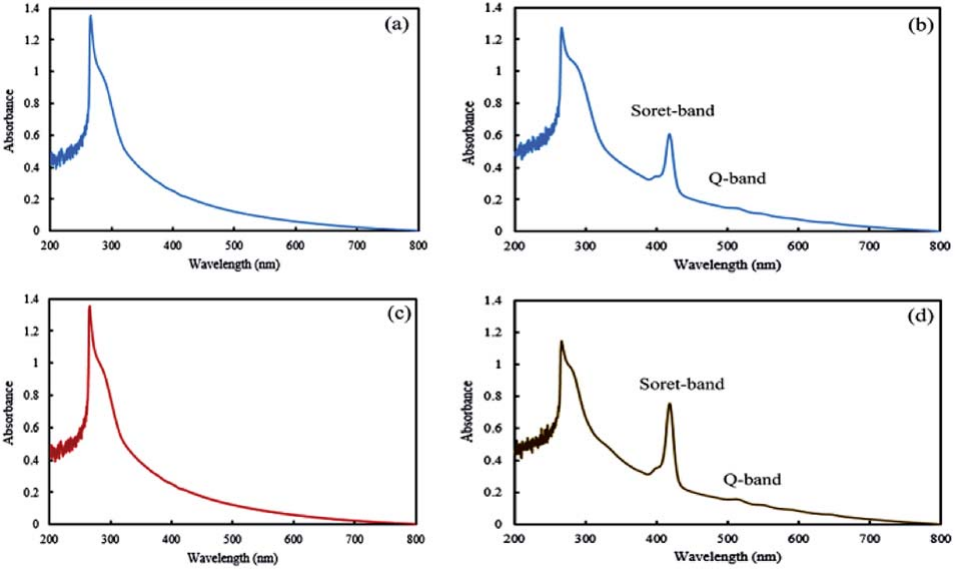
UV-Vis absorption spectra of mixed (a and b) and Janus (c and d) NPs before and after TPPC3-COOH grafting.

In the final step of preparing the mixed and Janus NPs, the TPPC3-COOH photosensitizer was grafted to the existing copolymers in J-(PCL-GO-copolymer) and m-(PCL-GO-copolymer) (Scheme 1). After the removal of the redundant TPPC3-COOH using the Soxhlet extractor, a UV-Vis spectrophotometer was utilized to confirm the successful attachment of TPPC3-COOH to poly-(NIPAm-co-NMA) in the mixed and Janus NPs. Before grafting TPPC3-COOH to the NPs, the UV-Vis absorption of the mixed and Janus NPs displayed a peak at 270 nm, which is related to graphene oxide (Fig. 5a and b), while an intense peak placed at 420 nm (Soret band) and a less intense peak at 530 nm (Q band) were observed in the absorption spectrum of the TPPC3-COOH grafted mixed and Janus NPs (Fig. 5c and d).

**Fig. 6:**
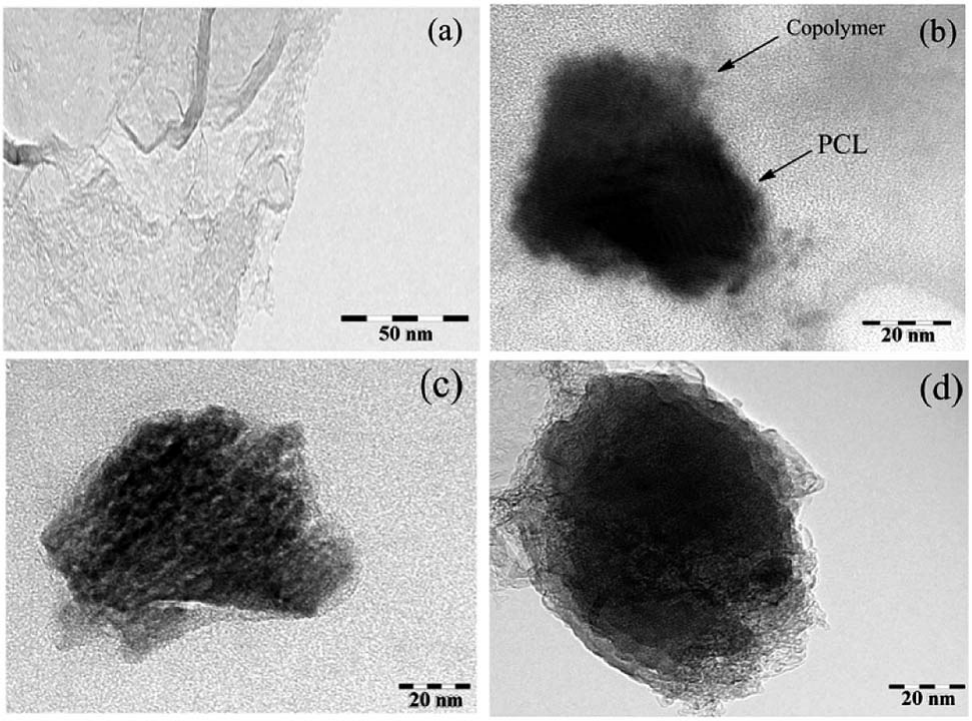
TEM micrograph of GO (a), J-(PCL-GO-copolymer) (b), and m-(PCL-GO-copolymer) (c and d) (at a magnification of 200, 150, 250 and 300k×, respectively).

The morphology of the final mixed and Janus NPs was investigated by transmission electron microscopy (TEM) and scanning electron microscopy (SEM). TEM images illustrated in Fig. 6 revealed different morphologies for GO, mixed, and Janus NPs. According to Fig. 6a, the GO sheets had flat surfaces and wrinkled edges. J-(PCL-GO-copolymer) NPs depicted a phase separation. In this case, PCL and copolymer chains were separately grafted on the GO surface with a regular pattern and distinct boundary (Fig. 6b). Nevertheless, in the mixed NPs, polymers were grafted haphazardly with an irregular pattern (Fig. 6c and d). These results confirm that the Pickering emulsion-free emulsifier method had been applied successfully to produce Janus NPs, where GO acted as the surfactant.

The different morphologies of the synthetic NPs were also revealed through SEM. Janus NPs displayed a compact agglomeration (Fig. S8a†), whereas a separated morphology appeared for the mixed ones (Fig. S8b†). There are certain reasons why Janus NPs adopt such behavior. First, the hydrophobic interactions of the PCL chains lead to the self-assembly of NPs, making up a micro-sized core.^43^ Second, the copolymer chains of several Janus NPs settle on the periphery of the macrocluster to optimize the repulsive interactions.

**Fig. 7:**
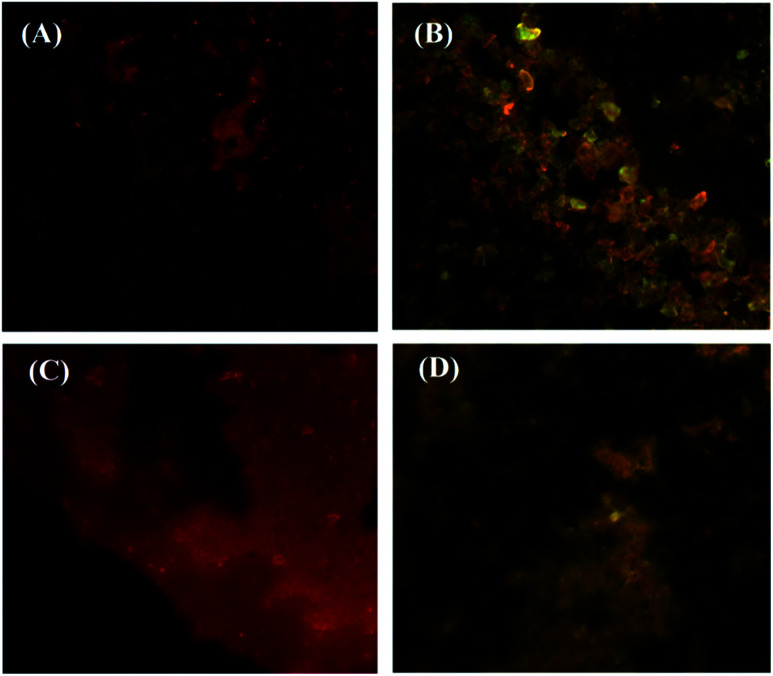
Fluorescent images of Janus (A and B) and mixed (C and D) nanoparticles under violet (A and C) and blue (B and D) beams.

Fig. 7 shows fluorescence microscopy images of Janus (A and B) and mixed (C and D) nanoparticles irradiated with violet (380–450 nm) and blue (450–495 nm) beams. As can be seen from the images, the green and red colors are related to fluorescein and acridine orange, respectively, which are located in both NPs. According to Fig. 7A, some brilliant red domains are observable, which are related to the excited acridine concentrated in a half portion of the Janus NPs. However, these Janus NPs under a blue beam show both colors in separated domains (Fig. 7B). In contrast, when the mixed NPs were irradiated with blue and violet beams, a scattered red pattern was illustrated under the violet light due to the presence of acridine (Fig. 7C). The appearance of several borderless domains in red and green colors under the blue light irradiation confirmed the validity of the synthesis of mixed NPs (Fig. 7D).

### The evaluation of ^1^O_2_ generated by porphyrin-grafted Janus and mixed NPs

**Fig. 8:**
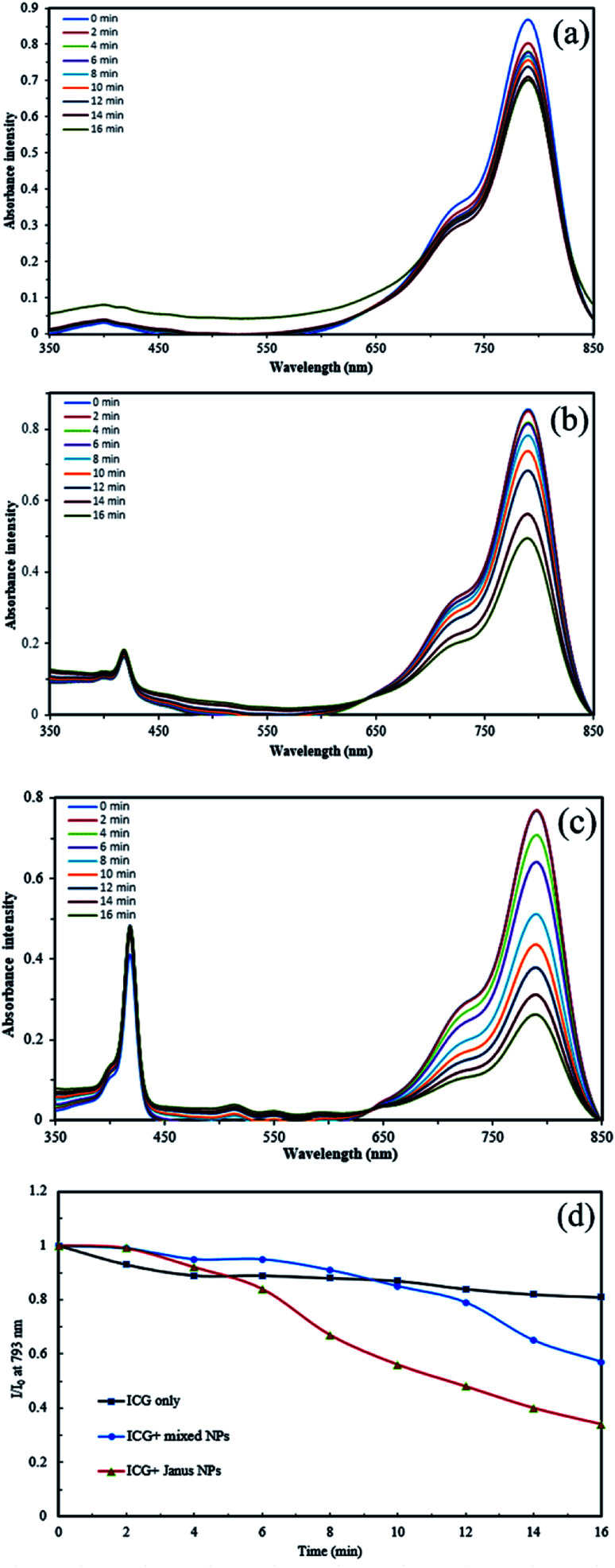
Time-dependent absorption spectra of (a) ICG alone, (b) ICG in the presence of porphyrin-grafted m-(PCL-GO-copolymer) NPs, (c) ICG in the presence of porphyrin grafted J-(PCL-GO-copolymer) NPs, and (d) decomposition rate of ICG alone, ICG in the presence of the mixed and Janus NPs under irradiation.

The ability of UV-light irradiated NPs to produce singlet oxygen was studied with the help of indocyanine green (ICG) as a probe and time-dependent absorption spectroscopy. Fig. 8 depicts the UV absorption spectra of ICG blank and Janus and mixed NPs in the presence of ICG under irradiation. Based on Fig. 8a, the ICG absorption at 793 nm under irradiation had a small decrease. However, the absorbance of ICG in the presence of the porphyrin-grafted mixed and Janus NPs under irradiation had a steady decrease over time, which is related to the decomposition of ICG by ^1^O_2_ generation under irradiation (Fig. 8b and c). In addition, Fig. 8d presents a comparison between the decomposition rates of ICG alone and ICG in the presence of both NPs under irradiation, in which *I*_0_ and *I* are the ICG absorbance at 793 nm in the initial time (*t* = 0) and various times, respectively. ICG absorption in the ICG blank maintained 80% of its initial absorption, while in the mixed and Janus NPs, it reached 57% and 34%, respectively. The reason why Janus NPs produce more ^1^O_2_ than the mixed ones is related to the amount of copolymer grafted on the surface of the GO NPs. According to the TGA curves of the mixed and Janus NPs, the percentage of the copolymer in the Janus NPs is more than that of the mixed ones. As a result, there is more TPPC3-COOH grafted to the Janus NPs. Therefore, the decomposition rate of ICG is effectively higher in the presence of Janus NPs than in the mixed ones.

### Preparation and investigation of drug-loaded Janus and mixed NPs

Quercetin, a hydrophobic anti-cancer drug with two benzene rings, was used for loading into both NPs through the drug-trapping method. Table 2 gives the percentages of drug loading and the encapsulation efficiency of both Janus and mixed NPs with a 10 wt% drug-to-NP ratio. According to the results of UV-Vis spectroscopy, the mixed NPs have a higher EE% than that of the Janus ones. Quercetin has the ability to aggregate in the hydrophobic parts of NPs because of its hydrophobic nature. Moreover, it is capable of forming hydrogen bonds with the hydrophilic parts of the NPs. It seems that the interface of PCL (hydrophobic section) and copolymer (amphiphilic section) is the best position for the loading of quercetin. As a result, Janus NPs with fewer hydrophilic/hydrophobic interfaces depicted a lower DL and EE% than that of the mixed ones. Dynamic light scattering (DLS) data showed that the mean hydrodynamic diameter of the NPs after quercetin loading increased in a water solution. Although the zeta potential of the drug-loaded NPs declined, the colloidal suspension of NPs had desirable stability (Table 2).

**Table 2:** Physical properties of NPs with and without drug

Nanoparticles	Janus		Mixed	
	Without drug	With drug	Without drug	With drug
Drug/nanoparticle in feed (%)	—	10	—	10
Drug loading (%)	—	6.5	—	7.9
Encapsulation efficiency (%)	—	72.3	—	86.9
Size (nm)	175 ± 4.6	179.9 ± 3.58	152 ± 3.36	161 ± 5.08
PDI	0.55	0.42	0.24 ± 0.04	0.31 ± 0.04
Zeta potential	+15.1	+19	+17 ± 0.80	+22.5 ± 1.04

**Fig. 9:**
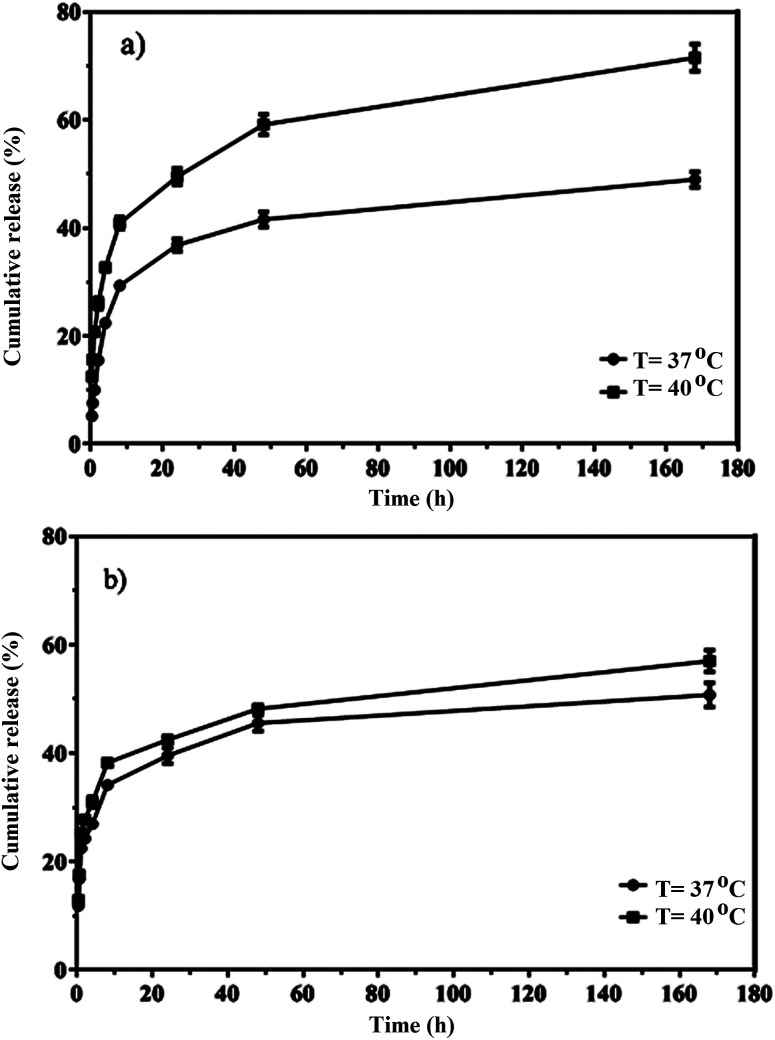
*In vitro* release of quercetin from porphyrin-grafted Janus (a) and mixed (b) NPs.

The *in vitro* release of quercetin from the mixed and Janus NPs was conducted in PBS at 37 and 40 °C and a pH value of 7.4. Thermo-sensitive polymers were designed to release the loaded drug at the temperature of cancerous cells (above 37 °C). The drug release profiles of the mixed and Janus NPs were calculated at 37 (body temperature) and 40 °C to assess the sensitivity of these NPs. Based on Fig. 9, the release curves of both types of NPs were higher at 40 °C than 37 °C. In addition, compared to our previous work,^44^ Janus NPs were more sensitive to temperature changes than the mixed ones due to the lower interaction between PCL and copolymer in the Janus NPs.

**Fig. 10:**
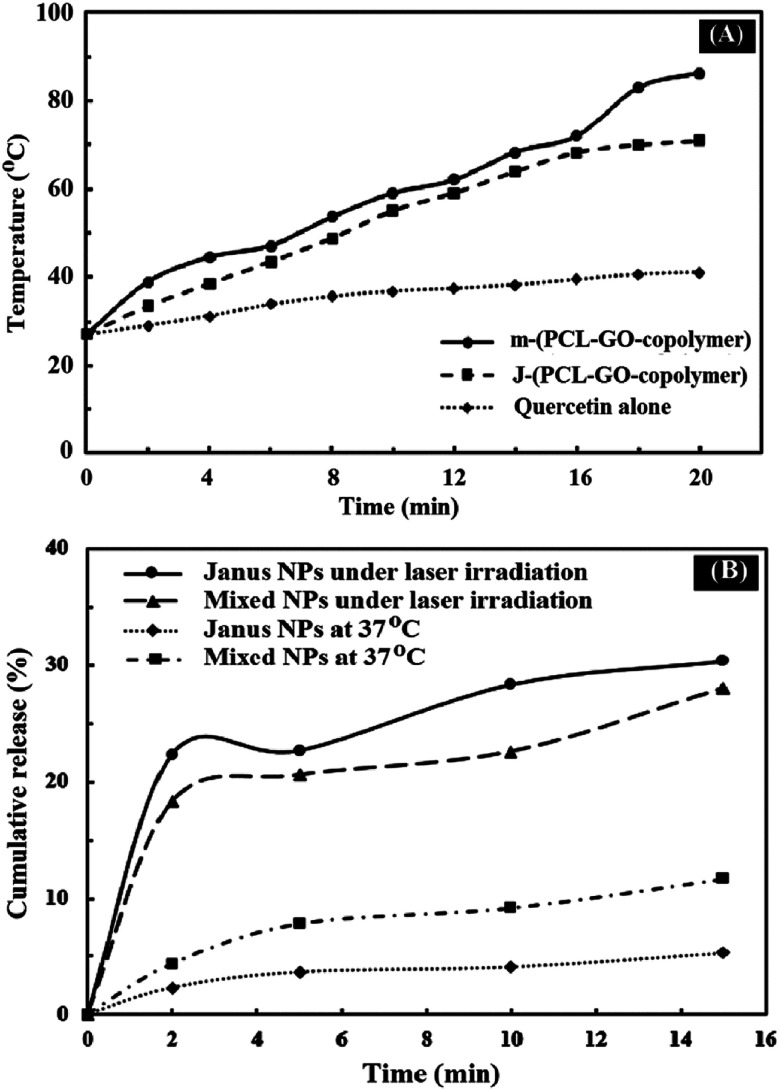
Photothermal capacity of quercetin, mixed and Janus NPs (A) under irradiation with 808 nm laser for 20 min; NIR-triggered release of quercetin from porphyrin-grafted Janus and mixed NPs with a temperature change under NIR irradiation (2 W cm^−2^) for 15 min and at 37 °C using a heater stirrer for 15 min (B).

Graphene and graphene oxide are capable of converting NIR radiation into vibrational energy and heat.^45^ As shown in Fig. 10A, the temperature of J-(PCL-GO-copolymer) and m-(PCL-GO-copolymer) NPs increased rapidly from room temperature to 70.9 and 86.3 °C, respectively. In comparison, there were no significant changes in the temperature of quercetin alone under NIR laser radiation. These results show that the synthesized mixed and Janus NPs can be used effectively as a photothermal agent in photothermal therapy. In addition, the accelerated quercetin release of the drug-loaded mixed and Janus NPs was measured through the photothermal effect of the Janus and mixed NPs under NIR laser irradiation (808 nm, power density of 2 W cm^−2^, and spot size of 2 cm^2^). Based on Fig. 10B, a burst release of quercetin was observed from the Janus and mixed NPs in 15 min, which is significantly higher than the cumulative release of quercetin from these NPs at 37 °C at the same time without NIR laser irradiation. Also, it is clear that the Janus NPs were more effective than the mixed ones because the release profile of the Janus NPs at 37 °C was lower than that of the mixed ones without laser irradiation, while the release profile of quercetin in the Janus NPs significantly increased under laser irradiation. These results indicate that the NIR irradiation on NPs was transformed into vibrational energy to produce sufficient heat to increase the release profile of quercetin in both NPs.

### Cytotoxicity tests

**Fig. 11:**
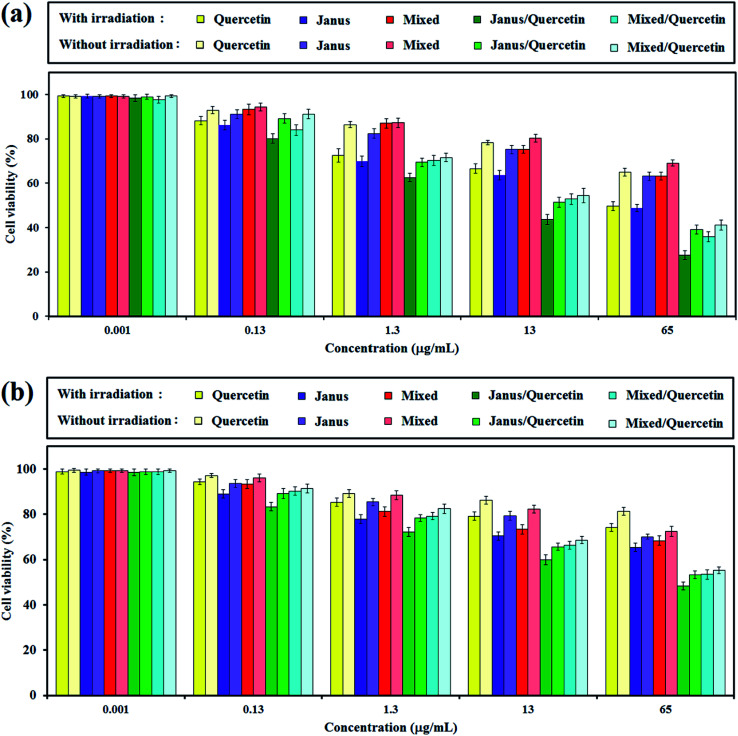
Viability of C6 glioblastoma (a) and OLN-93 (b) cells evaluated by the MTT assay with (10 min) and without light illumination after treating with quercetin, mixed and Janus NPs, and quercetin-loaded mixed and Janus NPs at different concentrations.

The *in vitro* cytotoxicity of free quercetin, mixed and Janus NPs, as well as quercetin-loaded mixed and Janus NPs with and without light illumination, were tested *via* an MTT assay against C6 glioblastoma and OLN-93 cells. Fig. 11a shows the viability of the incubated cancerous cells with the mentioned samples for 24 h. It is clear that the porphyrin-grafted mixed and Janus NPs exhibited a low dark cytotoxicity, even at a maximum concentration of 65 μg mL^−1^, and they could be safely used for drug delivery systems. In comparison with empty NPs, the quercetin-loaded mixed and Janus NPs show more dark toxicity, especially at higher concentrations.

The phototoxicity of the mentioned samples was then confirmed *via* light irradiation with a halogen lamp (500–800 nm, 70 mW cm^−2^) for an irradiation time of 10 min. According to Fig. 11a, the smaller phototoxicity of the mixed NPs compared to the Janus ones might correlate with the lower grafting of porphyrin in the mixed NPs, which caused insufficient photo-activity and generates less singlet oxygen. In addition, the quercetin-loaded mixed and Janus NPs exhibit potential phototoxicity over all samples. The results indicate that the synergistic effect of a combined chemotherapeutic drug and PDT is acceptable and can be considered as a favourable result. The phototoxicity of quercetin-loaded Janus NPs against cancerous cells was about identical to other reported work with nanoparticles containing π–π interactions between GO and porphyrin.^45^

The inhibitory effect of different nanoparticles on cell proliferation of OLN-93 as normal cells was performed similarly to those for C6 cells. According to Fig. 11b, the dark toxicity of quercetin-free Janus and mixed NPs on normal and cancerous cells is approximately identical. Quercetin was more toxic to cancerous cells compared to normal cells in dark conditions.^46^ When the concentration of NPs increased, the cell viability of OLN-93 dwindled steadily, especially when irradiated by the halogen lamp. And yet, the inhibition of cancer cell proliferation was greater than those of normal ones.

## Conclusions

In this work, we synthesized a thermo-responsive NIPAm-based copolymer by the ATRP method and a biocompatible PCL through a ring-opening polymerization. An amphiphilic copolymer and hydrophobic PCL were used to prepare polymer-modified graphene oxide NPs with mixed and Janus morphologies. Two-face Janus NPs were prepared with the Pickering emulsion method of o/w. Then, TPPC3-COOH was grafted onto the copolymer-grafted mixed and Janus NPs. We studied the different characteristics of quercetin-loaded NPs, such as size, morphology, drug capacity, and *in vitro* release. It was found that Janus NPs exhibit different behaviors in the TGA curves and TEM images. Furthermore, mixed NPs depict a higher quercetin loading than the Janus NPs due to the higher number of polymer–polymer boundaries in the mixed NPs, which is a suitable environment for trapping quercetin. However, the drug release studies revealed that Janus NPs are more sensitive to temperature changes from 37 to 40 °C, making this NP suitable for drug delivery systems in the human body. Also, the *in vitro* release of quercetin under laser irradiation was studied and the results showed that the drug release profile in both NPs was significantly impacted by laser radiation. Phototoxicity and dark toxicity tests demonstrated that the quercetin-loaded Janus NPs have excellent promise in PDT applications.

## Conflicts of interest

There are no conflicts of interest.

## Supplementary Material

RA-009-C9RA06058H-s001
