# Supplementary material for: An NIR-triggered drug release and highly efficient photodynamic therapy from PCL/PNIPAm/porphyrin modified graphene oxide nanoparticles with the Janus morphology
Source: RSC Adv. 2019 Dec 2;9(68):39780–92. doi: 10.1039/c9ra06058h (PMC9076064; doi:10.1039/c9ra06058h)
Supplement: RA-009-C9RA06058H-s001 [file RA-009-C9RA06058H-s001.pdf]

Electronic Supplementary Material (ESI) for RSC Advances.
This journal is © The Royal Society of Chemistry 2019

Supporting Information

NIR-triggered drug release and high efficient photodynamic therapy from PCL/PNIPAm/Porphyrin modified graphene oxide nanoparticle with Janus morphology

Sepideh Khoee*, Amirhossein Sadeghi

Polymer Laboratory, School of Chemistry, College of Science, University of Tehran, Tehran, Iran, PO Box 14155 6455

*** Corresponding author**

Characterization and equipment

^{1}H NMR (Bruker, 500 MHz) and Fourier-transform infrared spectroscopy (Bruker-Equinox 55 FT-IR) were used for the characterization of grapheme oxide, polymer, and porphyrin. The ^{1}H NMR spectrum was recorded in $CDCl_3$ to confirm the porphyrin derivations' structure. Also, ^{1}H NMR spectroscopy was applied to determine the structure and number average molecular weight (Mn) of PCL and copolymer in $CDCl_3$ and D_2O, respectively. The lower critical solution temperature (LCST) of the copolymer was studied by ultraviolet-visible light (UV-vis) spectrophotometer (Jasco-v 750) coupled with a temperature controller. A solution of polymer in distilled water was prepared and the heating rate was programmed at 1 ℃/min. Thermal gravimetric analysis (TGA) was performed to show the percentage of polymer grafted on the surface of graphene oxide (TGA Q600 V20.9 Build 20). Samples were heated at the rate of 10 ℃ per min under argon atmosphere, and the data were measured at the temperature interval of 20 to 600 �C. The morphology of GO and modified GO was studied by transmission electron microscopy (TEM) (JEM-2100 JEOL) at 200 kv accelerating voltage and also scanning electron microscopy (SEM) (FE¬SEM, Zeiss SuperATM 55, Germany). Janus and mixed nanoparticles were also tagged by a kind of gradual solvent-evaporation method using two different fluorescent dyes. Briefly, Acridine oargne and Fluorescein as organic probes were dissolved separately in acetone to prepared solutions with concentration of 1 mg/ml. Then, 1 mg of Janus NPs were dispersed in acetone and then 200 µL of dye solutions and distillated water (2mL) were added to nanoparticle suspension. The mixture was stirred for 4 h and then kept in the vacuum oven overnight (35). The average hydrodynamic diameter and zeta potential of NPs were estimated by dynamic light scattering (DLS) (HORRIBA) at 25 ℃. Furthermore, UV-vis spectroscopy was employed to investigate the release study of quercetin from NPs and used to confirm the grafting of TPPC3-COOH to Janus and mixed NPs. A chemical

method using ICG (indocyanine green) was applied to assess the capability of porphyrin-grafted mixed and Janus NPs in singlet oxygen generation. At first, a mixture of 80 µL of colloidal suspension of porphyrin-grafted Janus NPs in DMF (1 mg/mL) and 10 µL of ICG water solution (1 mg/mL) was added into DMF (10 mL) and irradiated by UV light source (360 nm, mW/cm^2). Then, UV-vis spectroscopy was utilized to detect the absorption intensity of the solution every two minutes. The same procedure was used for mixed NPs to assess the ability of singlet oxygen generation under irradiation.

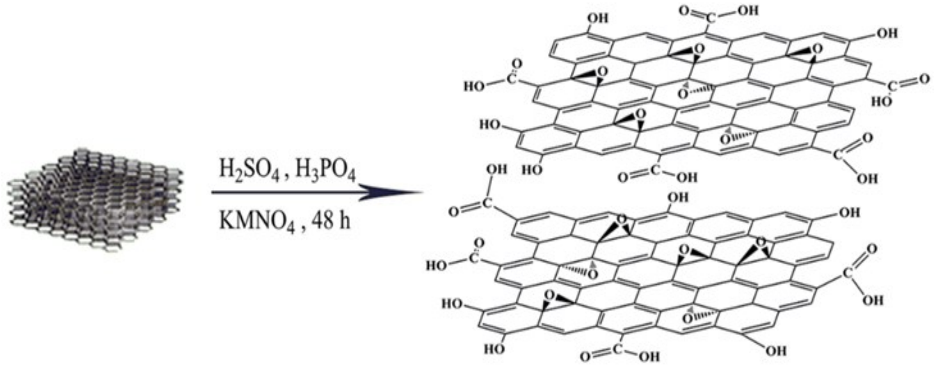

Scheme S1. Schematic representation of the synthesis pathway of GO

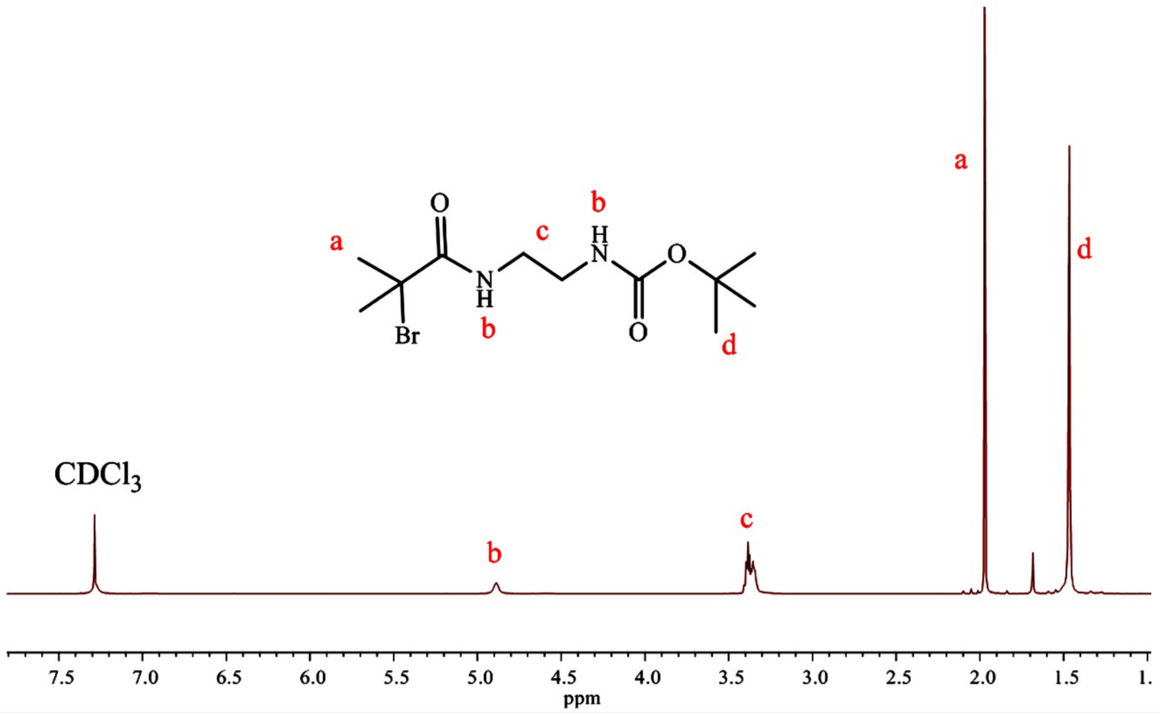

Figure S1. ^{1}H-NMR spectrum of tert-butyl 2-(2-bromo-2-ethylpropanamido)ethylcarbamate in $CDCl_3$

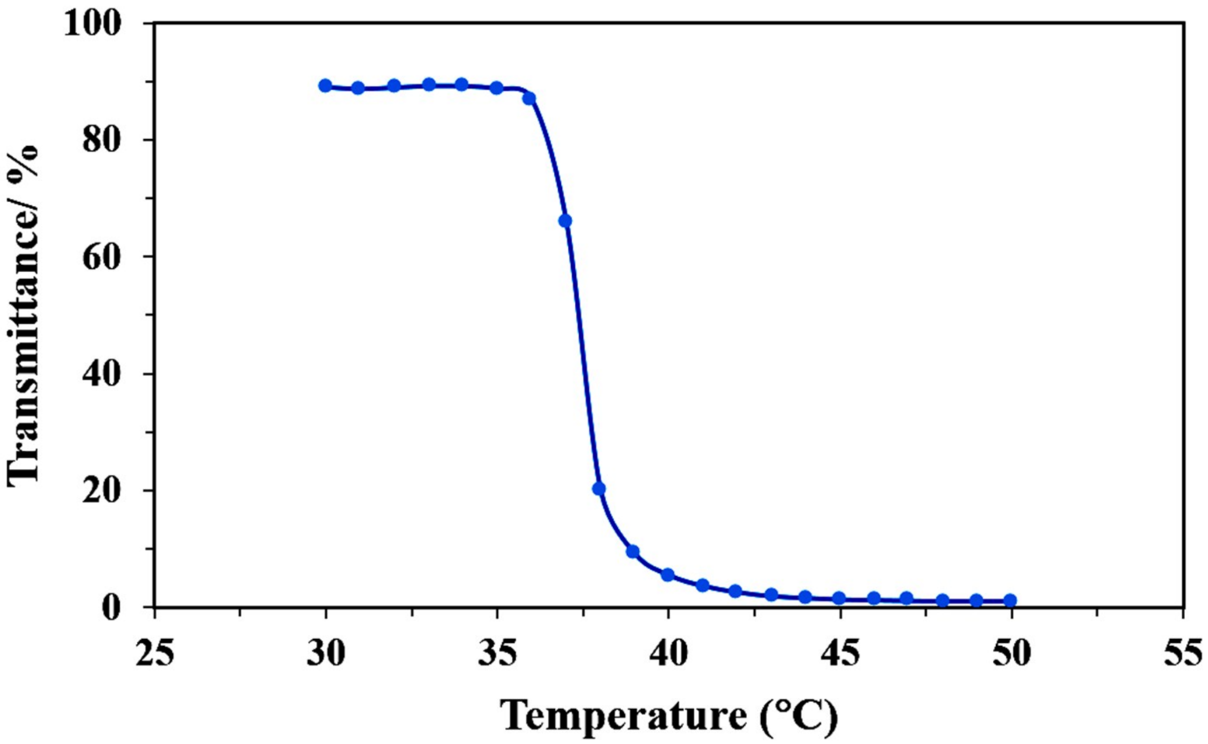

Figure S2. Temperature-dependent transmittance of p(NIPAm-co-NMA) in water

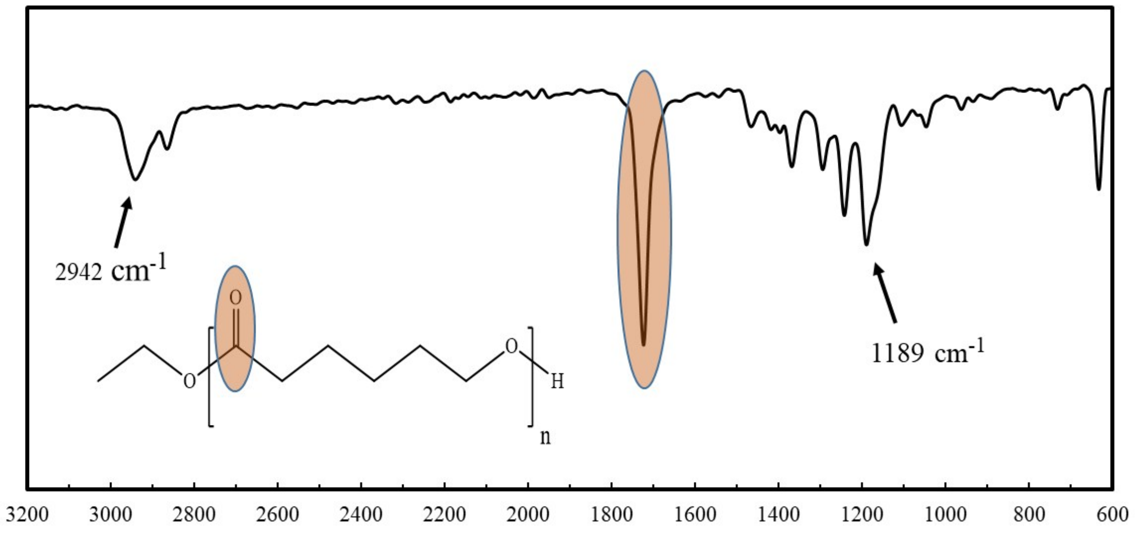

Figure S3. FT-IR spectrum of PCl

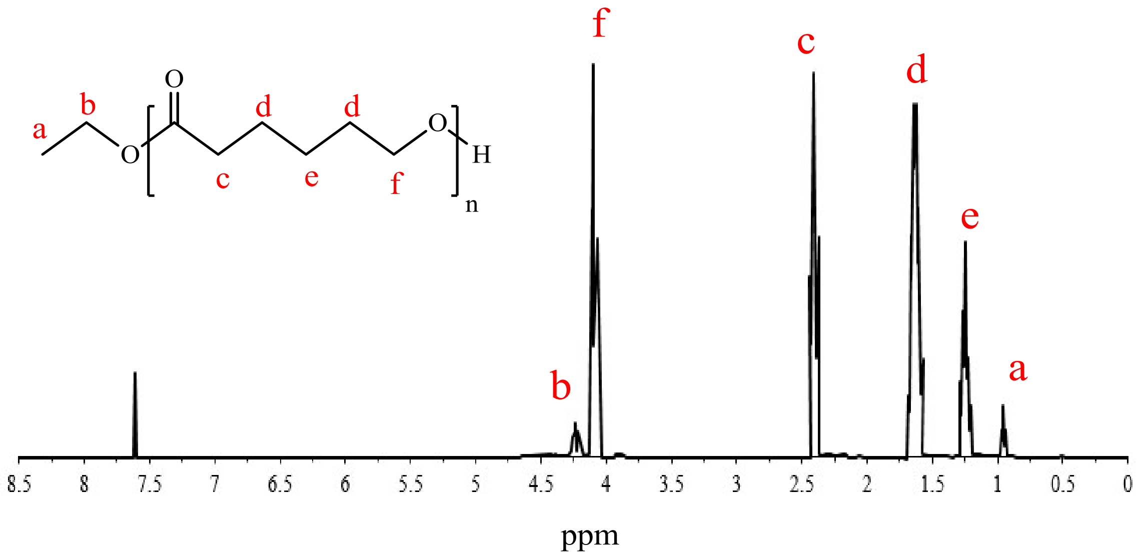

Figure S4. ^{1}H-NMR spectrum of PCl in $CDCl_3$

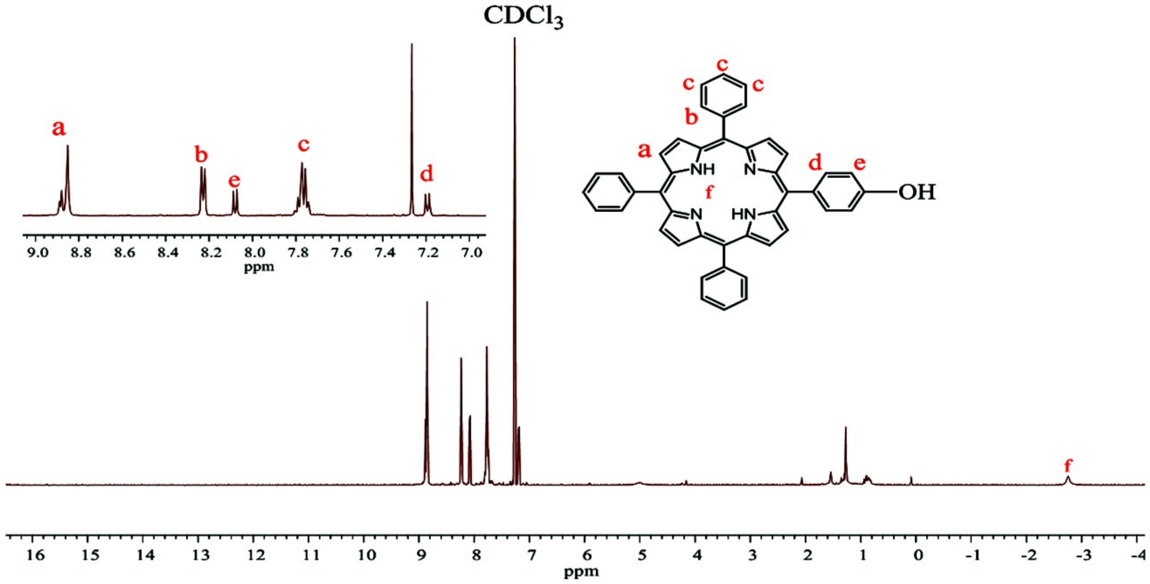

Figure S5. ^{1}H NMR spectrum of TPP-OH in $CDCl_3$

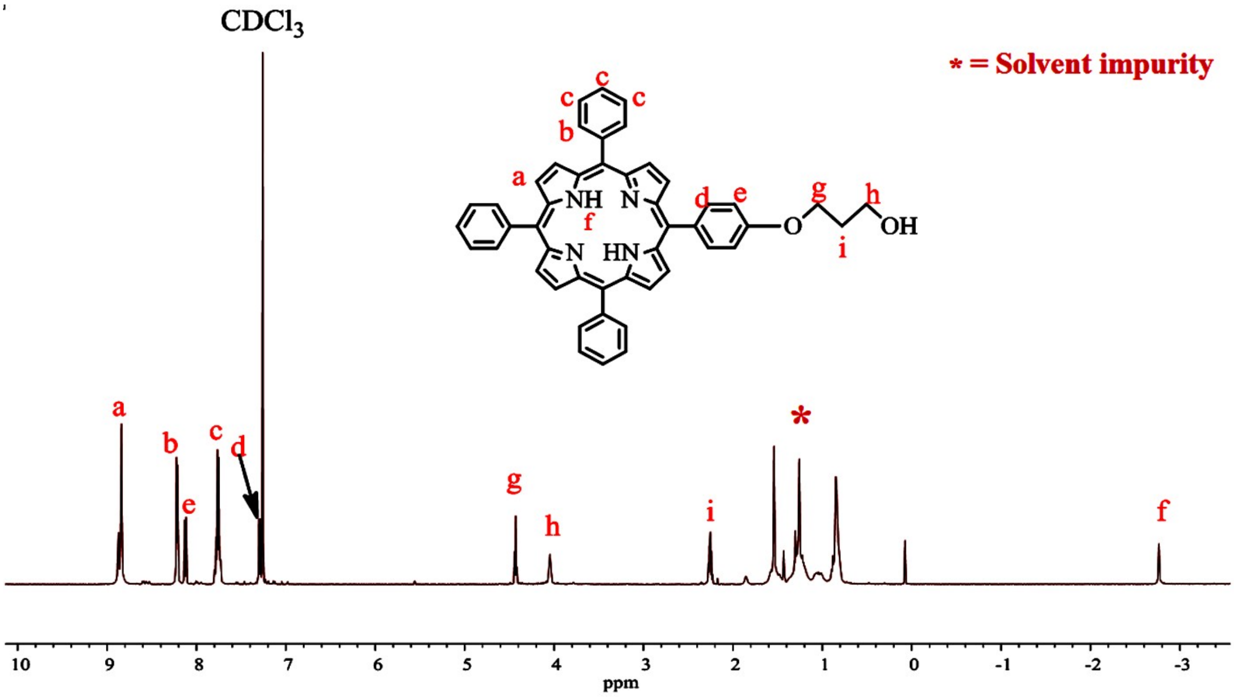

Figure S6. ^{1}H NMR spectrum of TPPC3-OH in $CDCl_3$

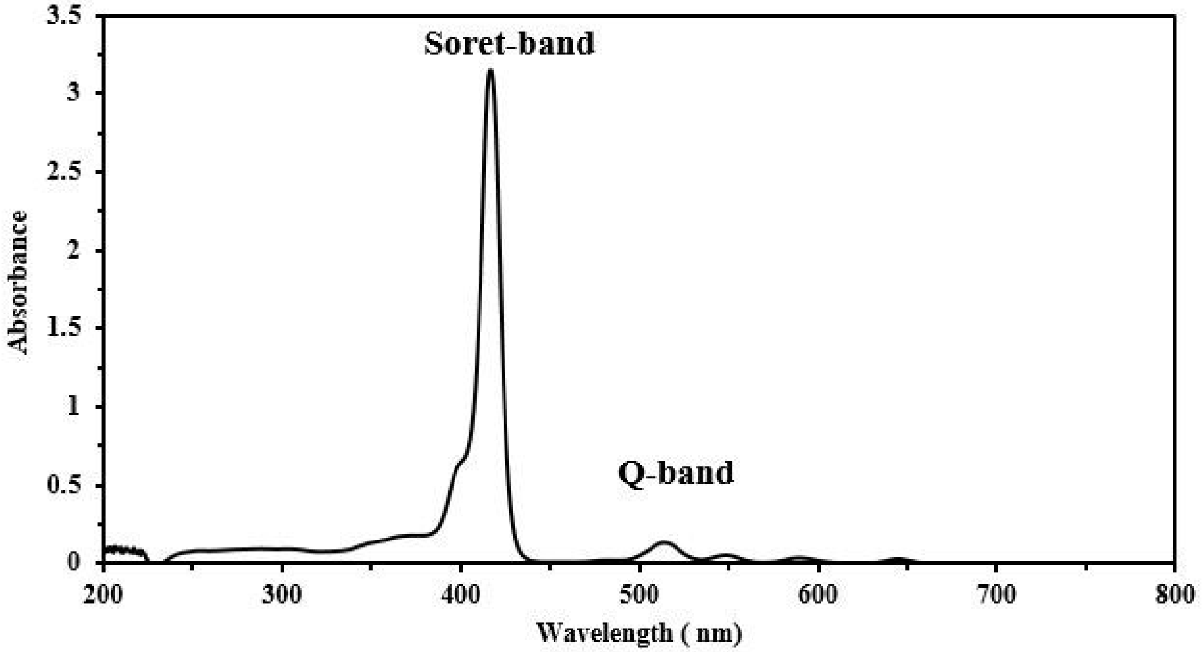

Figure S7. UV-Vis absorption spectra of TPPC3-COOH

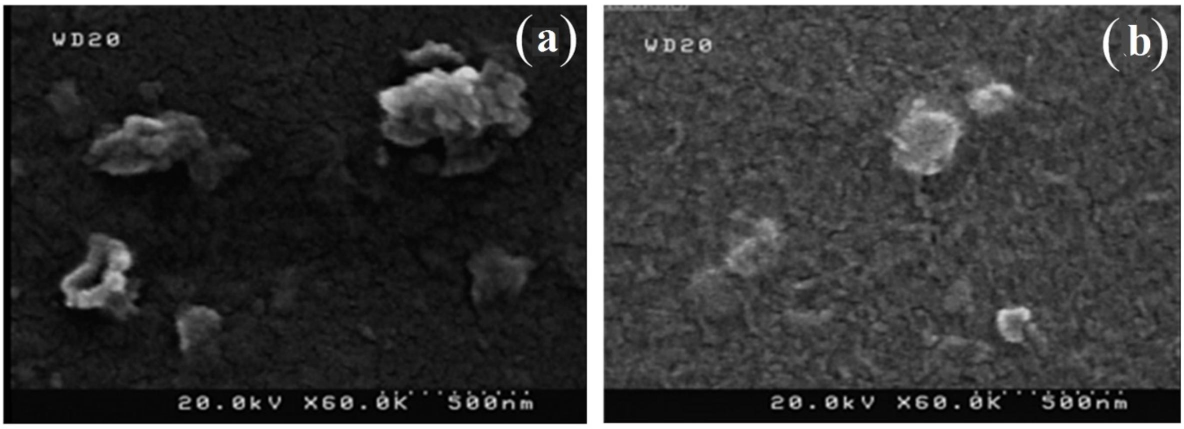

Figure S8. SEM images of Janus nanoparticle (a), and mixed nanoparticle (b)
